# Semi-automated biobank sample processing with a 384 high density sample tube robot used in cancer and cardiovascular studies

**DOI:** 10.1186/s40169-015-0067-0

**Published:** 2015-08-14

**Authors:** Johan Malm, Henrik Lindberg, David Erlinge, Roger Appelqvist, Maria Yakovleva, Charlotte Welinder, Erik Steinfelder, Thomas E Fehniger, György Marko-Varga

**Affiliations:** Section for Clinical Chemistry, Department of Translational Medicine, Lund University, Skåne University Hospital Malmö, 205 02 Malmö, Sweden; Centre of Excellence in Biological and Medical Mass Spectrometry, Biomedical Centre D13, Lund University, 221 85 Lund, Sweden; Department of Biomedical Engineering, Clinical Protein Science and Imaging, Biomedical Center, Lund University, 221 00 Lund, Sweden; Department of Cardiology, Lund University, Skåne University Hospital Lund, 221 85 Lund, Sweden; ThermoFisher Scientific, Glasgow, UK; First Department of Surgery, Tokyo Medical University, 6-7-1 Nishishinjiku Shinjiku-ku, Tokyo, 160-0023 Japan

**Keywords:** Biobank, 384 sample tubes, Cancer, Cardiovascular

## Abstract

**Background:**

In the postgenomic era, it has become evident that analysis of genetic and protein expression changes alone is not sufficient to understand most disease processes in e.g. cardiovascular and cancer disease. Biobanking has been identified as an important area for development and discovery of better diagnostic tools and new treatment modalities. Biobanks are developed in order to integrate the collection of clinical samples from both healthy individuals and patients and provide valuable information that will make possible improved patient care. Modern healthcare developments are intimately linked to information based on studies of patient samples from biobank archives in large scale studies. Today biobanks form important national, as well as international, networks that share and combine global resources.

**Methods:**

We have developed and validated a novel biobanking workflow process that utilizes 384-tube systems with a high speed sample array robot with unique processing principles.

**Results:**

The 384-tube format and robotic processing is incorporated into a cancer and cardiovascular diagnostic/prognostic research program with therapeutic interventions. Our biobank practice has gained acceptance within many hospitals and research units and is based on high-density sample storage with small aliquot sample volumes. The previous standard of 5–10 mL sample volume tubes is being replaced by smaller volumes of 50–70 μL blood fractions that typically result in hundreds of thousands of aliquot fractions in 384-tube systems.

**Conclusions:**

Our novel biobanking workflow process is robust and well suited for clinical studies.

## Background

Patient and society’s demand and expectations in health care combined with rising costs pose a great challenge on our health care system. The scientific community is expected to develop solutions that can improve clinical outcome and increase cost efficiency without jeopardizing the quality of care of individual patients. Future health care will be tailored to the individual and the introduction of personalized medicine in routine healthcare relies heavily on large scale biorepositories, biobanks (http://www.informatics-review.com/wiki/index.php/Biobanking_Definition) [1, 2].

Millions of clinical samples are collected every day for use in diagnostic tests that support clinical decision making. The majority of clinical routine samples are discarded but it is estimated that over one billion clinical samples are stored in biobanks worldwide [3]. The preservation of these samples is an important undertaking since each sample is unique and has the potential to facilitate future research and diagnostics.

Most errors in a hospital laboratory are due to preanalytical factors, if the preanalytical procedure fails subsequent downstream measurements will also fail [4, 5]. Preanalytical errors still account for 60-70% of all mistakes in laboratory diagnostics and in nearly one-fifth of these, errors might be associated with further inappropriate investigations and unjustifiable increase in costs [6].

The stability of a biospecimen sample depends on the method of storage. The vials must be sealed tightly, stored at the right temperature and the issue of possible freeze/thaw damage studied [7]. In almost all biobanks the samples are stored in 96-tube systems and each tube regularly used on more than one occasion. The current methods of sample handling and storage are often different in different laboratories, a fact that further complicates inter-laboratory studies, e.g. clinical multi-center studies.

Personalized medicine will be based on different diagnostic technologies used for studies of e.g. the human proteome. Results from proteome studies approaches [8–11**]** are important complements to genomic data [11–15] and provide crucial information about the target driver molecules and their post-translational modifications.

Quantitative mass spectrometry is an excellent way to identify biomarkers that can be used in personalized medicine. The technology is very sensitive and thus the sample quality is of utmost importance. We believe that new strategies must be advanced and established for the integration and use of biobank samples in routine health care, samples from patients that can be collected, processed and stored by a large scale archiving strategy.

We have previously reported on automated processing with robotic liquid handling for blood sample standardization developments for large scale biobanking [16, 17], as well as developments within standardized biobank workflows with evidence of sample history, tracking information, ensuring the sample integrity [18].

**Fig. 1:**
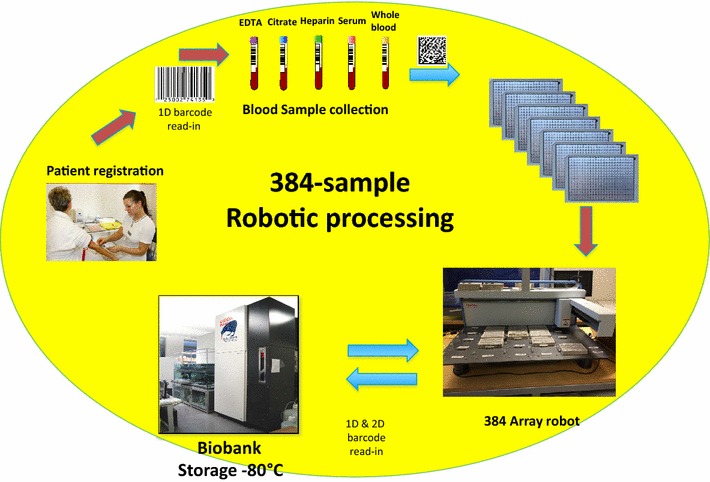
Biobanking work flow in the hospital where automated processing of 384-tube systems are processed by the “384 Sample Array Robot”.

We hereby present a semi-automated workflow for biobank samples utilizing the high density 384-tube system where the rigidity and performance of the automated platform is used within cancer and cardiovascular studies outlined in Fig. 1.

## Methods

### Liquid chromatography mass spectrometry

The peptide separation was carried out using a Thermo UPLC system (EASY nLC 1000, Thermo Scientific, San José, CA, USA). Aliquots of 0.5–1 µg of digested samples were injected onto a trap column (Acclaim PepMap100, C18, 5 µm, 100 Å, 300 µm i.d. ×5 mm, PN 160454, Thermo Scientific, San José, CA, USA). Peptides were then separated on an analytical column (Acclaim PepMap RSLC C18, 2 µm, 100 Å, 75 µm i.d. ×25 cm, nanoViper column, PN 164536, Thermo Scientific, San José, CA, USA) using a solvent gradient (0–40% acetonitrile in 90 min). Peptides separated on nanoLC system were analyzed with Q Exactive mass spectrometer is a positive ion mode. Samples were analyzed using an spray voltage and heated capillary temperature of 1.75 kV and 280°C, respectively. Data was acquired in a top ten data dependent acquisition mode (DDA), in which a high resolution 70,000 (at *m*/*z* 200)MS survey scan (*m/z* 350–1800, AGC value of 1e6, maximum injection time of 100 ms) was followed by an acquisition of tandem mass spectra (35,000 (at *m*/*z* 200), target AGC value of 1e6, maximum injection time of 120 ms). For the ten most intense ions, HCD fragmentation was performed and the MS/MS spectra were acquired. Dynamic exclusion was set to 20 s. The proteotypic peptides for each protein as well as the best transitions for each peptide are summarized in supplement.

### Sample array robot

The Sample Array Tube Handler (Thermo Scientific, San José, CA, USA) is specifically designed to be used with 384-tube plates. The platform is designed to hold up to 20 plates and can operate at lower temperatures (−20°C).

Tubes can be automatically transferred from a source plate (A) to a target plate (B) using two columns Excel file containing lists of tubes with source and destination positions. There are no requirements where to place the racks, all of the positions are open for both source and target plates.

The test was performed under three different conditions:

- At room temperature.
- At 5–8°C (cold room).
- At −20°C (freezer).

A large number of movements were made during the test period of the Sample Array robot.

We used several different picking profiles to check robot performance under all three conditions. When running the Sample Array robot in a freezer a web camera was installed together with a web interface to control the robot from outside.

## Results and discussion

### Preserving the integrity of clinical samples

Biobank sample integrity is paramount for subsequent studies but also other factors are important for showing the community that we are acting responsibly. Today’s clinical scientist needs to be prepared to not only collect and validate their raw gene-and protein- data and to safe keep the stored samples, but also to establish and maintain a legal administration for documenting the position of every individual sample in the biobank by a process that de-identifies the actual personal information linked to a specific sample. Currently the sample processing of blood samples from patients is performed at short cycle times within hospitals, typically 2 h [12–15]. The 2 h include the collection from the patient, sample handling, centrifugations, and aliquoting into 384 tube racks, i.e., from the time the needle goes into the arm until the aliquoted blood fractions are stored at −80°C. The workflow of the semi-automated 384-tube system processing and storage is depicted in Fig. 1. The protocol is used in cancer- and cardiovascular studies. In this workflow system, each 384 rack will hold one or two patients, depending on the number of primary tubes collected in the study. Here we typically use five primary tubes (6 mL) with blood containing various anticoagulants (EDTA, citrate, heparin). All blood fractions end up in the same rack due to the lack of time to distribute the aliquots into multiple 384-racks. This is the price that has to be paid, in order to manage handling blood samples from patients in a time cycle of less than 2 h.

**Fig. 2:**
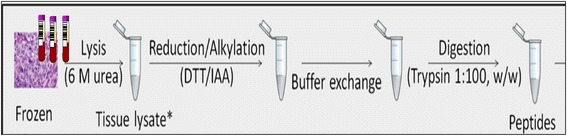
Protocol used for the sample preparation of clinical patient samples within biobank studies, using both tumor tissue and blood fractions.

Prior to protein expression analysis, the processing protocols are similar for both tissues from resected tumors from cancer patients, and blood samples from cardiovascular patients, as shown in Fig. 2. We have processed several series of sample cohorts in the sample work up protocols as outlined with high reproducibility and adaptability [10–12, 14].

**Fig 3:**
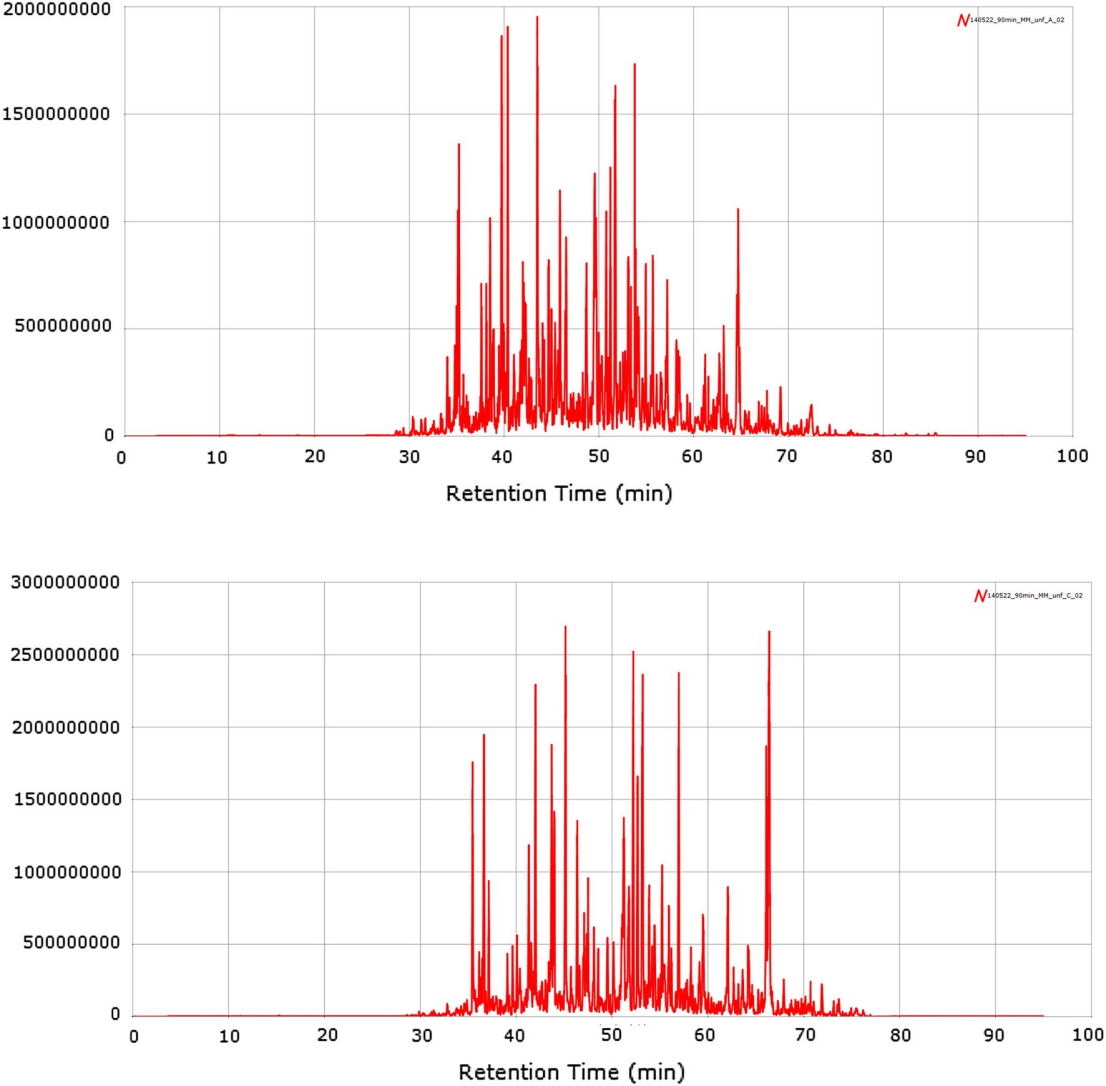
Resulting nano-chromatographic separation of biobank samples.

Proteomics analysis of biobank samples are currently performed in a large number of studies where the output with the latest high-resolution mass spectrometers is approx 100–150,000 protein sequences for a given tumor tissue sample and approx. 10–15,000 protein sequences analysed in blood fractions such as plasma or serum. Two examples are provided where protein sequence expressions are presented for two patients (see Fig. 3).

Most of the peptides are eluted during a cycle time of 40 min, if dead volumes are corrected for (30–70 min. These high resolution nano-chromatograms show extreme power, where the interface to the mass spectrometer is carrying out the second part of the separation along with the peptide annotation (nano LC–MS), followed by the peptide sequencing (nano LC–MS/MS).

This biobank workflow is currently utilized at the center of excellence in biological and medical mass spectrometry “CEBMMS” at Lund University in Sweden (http://cebmms.lu.se). The center is an investigator initiated activity with members in the Faculties of Medicine, Science, and Engineering, performing a number of clinical studies in collaboration with the Lund University hospitals.

### Automated processing of 384 sample tubes

Patient samples from the hospital were retrieved as outlined in Fig. 1, with the configured workflow steps being documented by barcode tagging. Frozen tissue sample processing was carried out as described in Fig. 2, where the solid tissue sections are homogenized, ending up as tissue extract solution samples. By this procedure, we are able to efficiently handle both biofluids and solubilized tissue extracts in the Sample Array Tube Handler system.

Automated sample processing is the only way to manage large sample numbers in sample archives where sample integrity is ensured [15, 16]. The increase in the number of high quality biobank samples results in an increased demand for the most appropriate sample type from a given sample set for proteomics and/or genomic studies. Large sample collections also need detailed clinical records matched that are matched to the sample being readily available.

Correspondingly, in order for us to maintain the sample integrity and quality over time in large scale biobanking, we apply the principle of single usage [17]. Single usage means, by our definition, that for any given stored sample in a biobank archive, it will be thawed once and never re-entered into the biobank for further storage. By this principle, we will ensure that sample integrity of each and every biofluid fraction or tissue is not variably changed by repeated thawing and freezing cycles. This consideration is especially relevant to large cohort studies where samples can easily reach thousands in number, thus we need controlled, efficient and automated workflows [18, 19]. The capacity of biobank sample collections is directly related to parallel automated processing. We are employing automated liquid handling instrumentations (HAMILTON robot MicroLab Starlet, Hamilton, Bonaduz AG, Switzerland) and high-density storage at −80°C (LICONIC freezer STT1k5 ULT, Liconic AG, Mauren, Liechtenstein). Currently, our ultralow robotic temperature storage facilities (−80°C), is able to store samples from clinical studies at a density of 5 million tubes using the 384-tube format, within a single freezer unit of approximately 3 m^3^ in volume.

In order to investigate the rigidity and stability of the Sample Array Tube Handler, we performed repeated picking and sorting steps in the 384 rack system. This was performed in an experimental setting using 4 different 384-racks filled with 384 separate sample tubes that were transferred over the platform board, from one end to the other.

The processing steps were performed according to the following protocol. A sample transfer scheme with specific XY positions was created in a method part of the Sample Array software. Tubes from the series of source plates (A) were then transferred to the destination series of plates (B) according to the designed scheme.

1. Open method part of the S384 software.
2. Design of the sample transfer with specific XY positions.
3. Pick up the sample tube from the A series of racks.
4. Deliver the sample tube from the A series of racks to the B series of racks.

Each step within the method is monitored and a corresponding CSV file is generated. An example of the data output file generated after transfer is presented in Table 1, where the X–Y coordinate of each sample tube transfered from A1, B1, C1 and D1 to the A2, B2, C2 and D2-384-tube plates are tracked.

**Table 1:** Output file from the Sample Array, the table builds on the worklist used and only adds information relevant for the process (columns I to N)

Tube ID	Name of source plate	Position on instrument	Tube position	Name of target plate	Position on instrument	Tube to be moved to position	Name of target plate	Position on instrument	Tube moved to position	Time and date start move	Time and date finished move
1	SOURCE-A1	A1	A1	DESTINATION-B1	B1	A1	DESTINATION-B1	B1	A1	04-05-2015 16:31	04-05-2015 16:32
	SOURCE-A1	A1	A2	DESTINATION-B1	B1	A2	DESTINATION-B1	B1	A2	04-05-2015 16:32	04-05-2015 16:32
	SOURCE-A1	A1	A3	DESTINATION-B1	B1	A3	DESTINATION-B1	B1	A3	04-05-2015 16:32	04-05-2015 16:32
	SOURCE-A1	A1	A4	DESTINATION-B1	B1	A4	DESTINATION-B1	B1	A4	04-05-2015 16:32	04-05-2015 16:32
	SOURCE-A1	A1	A5	DESTINATION-B1	B1	A5	DESTINATION-B1	B1	A5	04-05-2015 16:32	04-05-2015 16:32
	SOURCE-A1	A1	A6	DESTINATION-B1	B1	A6	DESTINATION-B1	B1	A6	04-05-2015 16:32	04-05-2015 16:32
	SOURCE-A1	A1	A7	DESTINATION-B1	B1	A7	DESTINATION-B1	B1	A7	04-05-2015 16:32	04-05-2015 16:32
	SOURCE-A1	A1	A8	DESTINATION-B1	B1	A8	DESTINATION-B1	B1	A8	04-05-2015 16:32	04-05-2015 16:32
	SOURCE-A1	A1	A9	DESTINATION-B1	B1	A9	DESTINATION-B1	B1	A9	04-05-2015 16:32	04-05-2015 16:32
				Columns F,G and H; from the input file, instructions for the instrument			Columns I, J and K; operations performed by the instrument				

The generated table comes with no head lines, these are added afterwards to make the table understandable for the reader.

The entire operation of 4 × 384 tubes is experimentally 1 cycle within our evaluation and validation of the S384 platform, integrated into a biobank environment. We have performed more than 100,000 single tube transfers on the platform with the protocol described above. The platform allows transfer of tubes from 10 source plates into 10 destination plates.

Each transfer step takes about 4.5 s, which results in a total experimental time of close to 13 h non-stop processing for transfer of 10,000 tubes.

We have performed sample transfer experiments, both within a day, in-between days and in-between weeks. In total we have processed more than 100,000 transfers over a 1 month period. We had no failures reported throughout these experiments over a 1 month period.The data was generated at ambient temperature. We also repeated the same experiments at −20°C and found the rigidity of the Sample Array platform to be very similar to that of the ambient performance. The resulting report file generated from each cycle with 10,000 sample tubes indicated no errors. Five cycles were studied.

The sample quality is maintained throughout the biobank handling processes which is illustrated in the resulting proteomics spectra shown in Fig. 3 where patient tissues were analysed by LC–MS using a Q Exactive platform.

### Sample tube distribution into multiple racks by scrambling

Large scale biobanks with millions of sample tubes need a given structure and organization in order to ensure the safety of the samples. In principle the sample archive should have a backup. In this respect, all sample tubes from a single patient should never be stored within one 384 rack. Instead, the sample tubes should be divided and spread into multiple plates. In addition, a backup of each sample collection, approx 10–20% should be stored physically in another center located elsewhere, in order to make sure that the samples will never be lost.

Scrambling of samples into multiple racks can easily be achieved with the Sample Array Tube Handler platform.

### Biobank space optimization and refilling the 384 racks

With multiple studies going on at the same time the sample flow in and out of the biobank is highly active. The samples in a biobank will shift over time as samples are being collected and exported to research groups, in addition to the steady growth of the number of samples. These dynamic events will require that sample storage capacity is optimised.

Upon sample retrievals from biobanks, sample tubes from different 384-racks will be partly empty. In addition, the constant patient recruitment with tens of thousands of sample tubes per month makes it necessary to re-fill the 384-racks. The 384 robotic platform is an efficient work horse to fulfil this imoportant task.

### 384-sample tube tightness

The tightness of the tube sealing, when processed with the robotic fine mechanics arm, was investigated with tubes containing blood samples in liquid form (23°C) as well as with frozen blood (−20°C), and solubilized tissue extracts. The blood fractions from cancer and cardiovascular patient samples were processed as described in Fig. 1. The sample tubes were investigated by microscope in order to look for any deterioration in the alumina film that covers the 384 tubes. A sample set of 1,000 tubes was investigated at ambient temperature, and we could not see any traces of harm or deterioration on these tube coatings. Resistance to leakages was also investigated. The 84 racks were placed in a swing out centrifuge and centrifuged for 10 min. Upon reinvestigation we examined the racks for any leakages caused by the centrifugation. None of the sample tubes containing plasma and erythrocyte blood fractions showed any trace of leakage, which was considered a confirmation of the excellent performance of the Sample Array Tube Handler.

### Laboratory information management system (LIMS)

**Fig 4:**
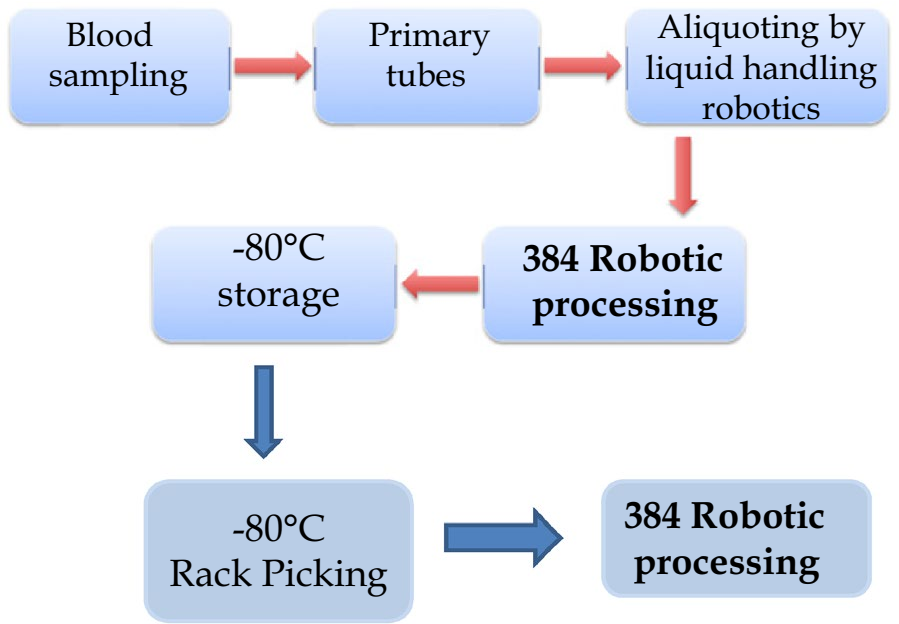
Schematic illustration of our work flow with electronic surveillance and LIMS integrated system.

The data handling process workflow of our biobank infrastructure is outlined in Fig. 4, where the 384 robotic processing plays a central role in the overall handling of patient samples. Consequently, the CSV-file generated by the Sample Array Tube Handler platform for each of 384 plates is automatically fed into the database through the laboratory information management system (LIMS). The new positions of each and every tube are aligned within the given study in the LIMS. The 2D barcode makes it possible to track each tube and position within the 384 rack. The clinical data that is associated with the sample tube of each patient is also linked to the CSV-file from the robot.

### Workflow advantages

The issue of poor biospecimen quality has often not been discovered until large number of clinical assays were performed or study reports already released [20]. The present system generates 70 µL aliquotes of whole blood (EDTA), plasma (EDTA, heparin, citrate), buffy coat (EDTA, heparin, citrate), red blood cells (EDTA, heparin, citrate) and serum in a 384-tube format using a fully automated sample processing strategy. This highly standardized procedure minimizes the probability of preanalytical errors and also makes possible the use of high quality samples on every occasion by avoiding the use of previously thawed samples.

The biobank practice can be implemented in clinical routine in hospital laboratories and has now gained acceptance within several health care units. Instead of larger 5–10 mL tubes or the 96-tube format the current system typically gives the biobank user access to hundreds of thousands of high quality small sample aliquots.

## Concluding remarks

The impact of personalized medicine with targeted treatments is increasing and it is not surprising that developing personalized medicine methodologies now conquer much of the current development portfolio within big pharma and the biotech industry. Translating basic science to discover novel and improved treatments is a huge challenge [21, 22]. Automated picking and sorting of samples in biobanks and sample repositories is a strategically important part of modern healthcare operations in order to advance our understanding and knowledge of the molecular and environmental basis of human diseases. The ultimate healthcare goal that forms the basis of large recent investments in biobanks is to provide the prerequisites for refined and improved diagnostics and treatments. This will eventually aid in developing personalized medicine and the understanding of systems biology.
